# Extraction Process Research and Characterization of Microcrystalline Cellulose Derived from Bamboo (*Phyllostachys edulis* (Carrière) J. Houz.) Fibers

**DOI:** 10.3390/polym17091143

**Published:** 2025-04-23

**Authors:** Zhu Liu, Zhongwei Wang, Shoulu Yang, Ning Ji, Dan Li

**Affiliations:** 1 Guizhou Academy of Forestry, Guiyang 550005, China; liuzhu9206@126.com (Z.L.);; 2 College of Material Science and Engineering, Central South University of Forestry and Technology, Changsha 410004, China

**Keywords:** bamboo, microcrystalline cellulose, acid hydrolysis

## Abstract

Microcrystalline cellulose (MCC) possesses important attributes, including high crystallinity, a large surface area, excellent mechanical strength, chemical stability, and biodegradability. This study aims to research MCC extraction from bamboo (*Phyllostachys edulis* (Carrière) J. Houz.) fiber by assessing the impact of key processing variables such as acid concentration, temperature, and hydrolysis duration. Experimental results indicate that hydrolysis time and hydrochloric acid (HCl) concentration significantly influence yield. After evaluating the effects of various hydrolysis conditions, the optimal parameters were determined to be a 2.0 M HCl concentration, 90 °C, and 10 min of reaction time. The MCC produced under optimal conditions displayed improved crystallinity (77.2%) while retaining functional groups similar to those found in raw bamboo. Morphological analysis revealed an irregular rod-like shape with rough surfaces. This optimized hydrolysis process offers a viable approach for MCC production from raw bamboo and holds potential as a precursor for developing environmentally friendly biodegradable fiber materials.

## 1. Introduction

Microcrystalline cellulose (MCC) is a purified, partially depolymerized cellulose derived from natural plant fibers through acid hydrolysis. It appears as white or nearly white porous particles, typically in a short, rod-shaped or powdery form, with β-1,4-glucosidic bonds. MCC is primarily produced through the hydrolysis of natural cellulose using dilute acid, resulting in a polymerization degree (LODP) ranging from 15 to 375. It is odorless, tasteless, and widely used in various applications due to its unique properties [1,2]. MCC can partially dissolve and swell in dilute alkaline solutions, exhibiting high reactivity in processes such as carboxymethylation, acetylation, and esterification. It remains insoluble in organic solvents like water, dilute acids, and oils. These properties make it highly advantageous for chemical modifications [3,4,5]. MCC, characterized by its low degree of polymerization and large specific surface area, is widely utilized in pharmaceuticals, food, cosmetics, and other light chemical industries [6,7,8,9,10]. Cotton and flax are the most popular sources of microcrystalline cellulose (MCC) due to their unique high cellulose content (90–95%), high crystallinity index (70–85%), and industrial advantages. Other common raw materials for MCC production include Ganteng residue and corn cob [11], beet pulp [12], wood pulp, rice husk [13], rice, and various plant shells [14,15] as well as other raw materials such as wheat, barley, peanut shells, etc., which contain high alpha cellulose. The process of producing MCC involves using dilute acid as a catalyst to hydrolyze the raw materials. After hydrolysis, the residue is washed, dried, and separated. Non-cellulose substances, such as lignin, are dissolved during this process, followed by additional washing, drying, and crushing steps to produce powdered MCC [16].

Bamboo, a fibrous material from Poaceae plants, primarily consists of fiber cells, parenchyma, and vessels, with fiber cells being the most crucial. As the fifth most abundant natural fiber after cotton, hemp, wool, and silk, bamboo fiber contains 44% to 53% cellulose. This high cellulose content makes it a valuable raw material for MCC production [17,18]. Zhang et al. [19] investigated the one-step synthesis of MCC from bleached Kraft bamboo pulp (BKBP) using low-concentration HCl (≤0.1 wt%). The impacts of hydrolysis parameters were analyzed, and the optimal conditions were determined as follows: 0.5 g of pulp, 0.8 wt% HCl, 165 °C, and 40 min. The study also investigated the morphological, physicochemical, and thermal properties of bamboo-derived MCC, highlighting its potential as a sustainable MCC source and a promising material for biocomposite packaging applications [20,21,22,23]. In composite material research, bamboo-derived MCC has been utilized as a bio-based carbon source in an intumescent system, enhancing the thermal stability, flame retardancy, and mechanical properties of polylactic acid (PLA) [24]. Masrat Rasheed et al. found that incorporating bamboo-derived MCC as an additive in a hot-pressed mixture of lactic acid (PLA) and butyl succinate (PBS) composites improved the interfacial bonding between PLA and PBS. Further, MCC enhanced the crystallinity, thermal stability, and tensile strength of the composites [25]. Therefore, bamboo-derived MCC holds significant potential as a reinforcing phase, particularly in enhancing mechanical properties [26,27].

While prior studies have explored microcrystalline cellulose (MCC) extraction from bamboo, most methods rely on harsh conditions, such as high-temperature hydrolysis (e.g., 165 °C) or prolonged reaction times (≥40 min) [19,23], which increase energy consumption and risk cellulose degradation. Additionally, existing protocols often prioritize yield over crystallinity or fail to systematically optimize critical hydrolysis parameters (acid concentration, temperature, time) for balancing efficiency and MCC quality. So, this study aimed to develop an efficient method for MCC preparation by investigating the isolation of MCC from a species of bamboo (*Phyllostachys edulis* (Carri è re) J. Houz.) fiber through hydrolysis. The effects of acid concentration, hydrolysis time, and temperature on extraction efficiency were evaluated to optimize the process and thus, introducing a low-temperature (90 °C), short-duration (10 min) process with moderate HCl concentration (2.0 M) to efficiently isolate MCC while minimizing energy use and preserving crystallinity. Similarly, the morphological and structural characteristics of the resulting MCC were analyzed to confirm with previous research.

## 2. Materials and Methods

### 2.1. Materials

The raw material was 5-year-old Moso bamboo (*Phyllostachys edulis* (Carrière) J. Houz.), harvested from Chishui City, Guizhou Province, China. Sodium chlorite (NaClO_2_, ≥78%), acetate (CH_3_CO_2_H, GR, 99.8%), Potassium hydroxide (KOH, AR, 85%), toluene (C_7_H_8_, ≥99.5%), ethanol (CH_3_CH_2_OH, AR) and hydrochloric acid (HCl, 37%) were procured from Shanghai Aladdin Industrial Inc. (Shanghai, China).

### 2.2. Methods

#### 2.2.1. Isolation of Cellulose

Cellulose was isolated from bamboo following the method described previously [28]. First, the raw bamboo was ground into a powder and passed through a 60-mesh sieve. Approximately 50 g of the powder was then dewaxed using a Soxhlet apparatus using 300 mL of a toluene/ethanol mixture (2:1, *v*/*v*) for 6 h. Sugar content, residual starch, and other impurities were eliminated through sequential chemical treatment. The purified sample was then delignified using sodium chlorite–acetic acid, following the method described by Wise et al. [29]. The sample was immersed in a sodium chlorite–acetic acid solution at 80 °C for 1 h, and this process was repeated three times. It was then subjected to a 2% *w*/*v* KOH solution at 80 °C for 2 h, followed by filtration and thorough washing with water.

#### 2.2.2. Preparation of Microcrystalline Cellulose

MCC was synthesized using the approach described by Ejikeme [30]. Approximately 20 g of the extracted bamboo cellulose underwent hydrolysis with HCl to break down its amorphous regions. The resulting MCC was then separated by filtration, rinsed with distilled water until a neutral pH was achieved, and subsequently dried in a tray dryer at 50 °C overnight. An orthogonal experiment was conducted to evaluate the influence of three key factors HCl concentration, temperature, and hydrolysis time on the isolation process, aiming to determine the optimal conditions. The experimental design, including the factor levels, is presented in Table 1 and Table 2. The yield of MCC was calculated using Equation (1). All of experiments were carried out in triplicate 3. The optimal level of experimentation was determined using range analysis in SPSS 27 software.

(1) $$\mathrm{Yield}\;(\%)=\frac{M_{1}}{M_{2}}\times 100\%$$

where *M*_1_ is weight of obtained MCC, *M*_2_ is weight of obtained bamboo cellulose.

#### 2.2.3. Characterization

##### FTIR Analysis

FTIR spectra were obtained using a Fourier transform infrared spectrometer to analyze the bamboo, bamboo fiber (BF), and bamboo MCC. The samples were crushed using a high-speed mill and passed through a 200-mesh sieve to obtain powder samples suitable for FTIR spectroscopy (Nicolet 670, Thermo Nicolet Corporation, Madison, WI, USA), and 1–2 mg of the powder sample was ground with potassium bromide (KBr) and mixed evenly. Then, transparent sheets were obtained using the tableting method. Each sheet was placed in the sample chamber of the FTIR instrument; the background scanning time was 16 s, and the wavelength range was 400–4000 cm^−1^.

##### SEM Analysis

Scanning electron microscope (SEM) images were captured with a ZEISS Sigma 300 (Carl Zeiss AG, Baden-Württemberg, Germany) scanning electron microscope with an accelerating voltage of 15 kV. All samples were coated with a gold paint conductive layer. The magnifications were ×200, ×400 and ×800. The size of the MCC particles was determined using Image-Pro Plus 6.0 software.

##### TGA Analysis

The thermal properties of the samples were assessed using TGA (Netzsch TG 209 F3, NETZSCH-Gerätebau GmbH, Free State of Bavaria, Baden-Württemberg, Germany). 5 mg of powder samples were used and the tests were conducted under either a nitrogen or airflow environment, with a flow rate of 30 mL/min, within a temperature range of 20–800 °C, and a heating rate of 10 °C/min. The data of the quality or rate of change in the sample with temperature during the heating process is recorded.

##### XRD Analysis

The X-ray diffraction (XRD) patterns were recorded using a Rigaku SmartLab SE X-ray (Rigaku Corporation, Tokyo, Japan) diffractometer operating at a voltage of 40 kV and a current of 30 mA. The scanning speed was set at 5°/min, and the 2θ range spanned from 5° to 60°. The degree of crystallinity (CI) was determined using Equation (2), which calculates the ratio of the crystalline peak area to the total area, where *I_total_* is the total area under XRD peaks (*I_total_* = *I_cry_* + *I_am_*), *I_cry_* is the XRD peaks at the crystalline region, and *I_am_* is the XRD peaks at the amorphous region. The Gaussian function in Origin 2021 was used for peak assignment of XRD images. The crystallinity and crystalline region size of samples were calculated based on X-ray diffraction patterns and peak assignment results. The crystalline region size was calculated based Equation (3), where D is the crystal layer spacing, *k* is the diffraction constant (0.89), *λ* is the incident wavelength (0.154 nm), β is the diffraction peak half width, and *θ* is the diffraction angle. The number of repetitions for crystallinity calculation was 3.

(2) $$\mathrm{CI}\;(\%)=\frac{I_{cry}}{I_{cry}+I_{am}}\times 100\%$$

(3) $$D=\frac{K\lambda }{\beta cos\theta }$$

##### Cellulose, Hemicellulose and Lignin Content Analysis

A total of 0.3 g of raw bamboo, BF and MCC samples, separately, were added to 3 mL of 72% sulfuric acid and reacted at 30 ± 3 °C for 60 min. After this, the reaction residues were transferred to a 250 mL glass bottle and washed with distilled water until completely filtered. The residues were dried in an oven at 105 °C for 4 h and weighed to calculate the mass of acid insoluble lignin (*M*_1_). The filtrates were collected to calculate the content of acid-soluble lignin (*M*_2_) and sugar by UV–visible spectrophotometry. Four percent sulfuric acid was used as a reference, absorbance was measured at 205 nm with a molar absorptivity of 113 L·g^−1^·cm^−1^, and soluble lignin content was calculated to obtain lignin content according to Equation (4). A total of 20 mL of filtrates were added into a 50 mL beaker and neutralized with CaCO_3_ to pH 5–6. High Performance Liquid Chromatography was used to test the content of glucose (*C*_*glucose*_), xylose, and arabinose (*C_xylose+arabinose_*), and the content of cellulose and hemicellulose is calculated according to Equations (5) and (6).

Lignin content (%) = *M*_1_ + *M*_2_ (4)

(5) $$\mathrm{Cellulose}\;(\%)=\frac{C_{glucose}\times 87\times 10^{-3}\times 0.9}{0.3}\times 100\%$$

(6) $$\mathrm{Hemicellulose}\;(\%)=\frac{C_{xylose+arabinose}\times 87\times 10^{-3}\times 0.88}{0.3}\times 100\%$$

##### Gel Permeation Chromatography (GPC) Measurements

To enable GPC analysis, BF and MCC samples were derivatized into cellulose tricarbanilate (CTC) through a phenyl isocyanate reaction. The procedure involved mixing 15 mg of dried cellulose with 4 mL of anhydrous pyridine and 0.5 mL of phenyl isocyanate in a sealed flask. The mixture was stirred at 70 °C for 48 h. After cooling, excess reagent was quenched with methanol, and the CTC product was precipitated by adding a methanol–water solution (7:3 *v*/*v*). The precipitate was purified via centrifugation and sequential washing with methanol–water and deionized water. CTC samples were dissolved in tetrahydrofuran (THF), filtered through a 0.45 μm polytetrafluoroethylene (PTFE) membrane, and transferred to autosampler vials. GPC measurements were performed using a Waters GPC 1515 system. Molecular weight parameters, including number-average molecular weight (*M_n_*) and weight-average molecular weight (*M_w_*), degree of polymerization (DP) were calculated.

## 3. Results and Discussion

### 3.1. Effects of Process Variables on MCC

**Figure 1:**
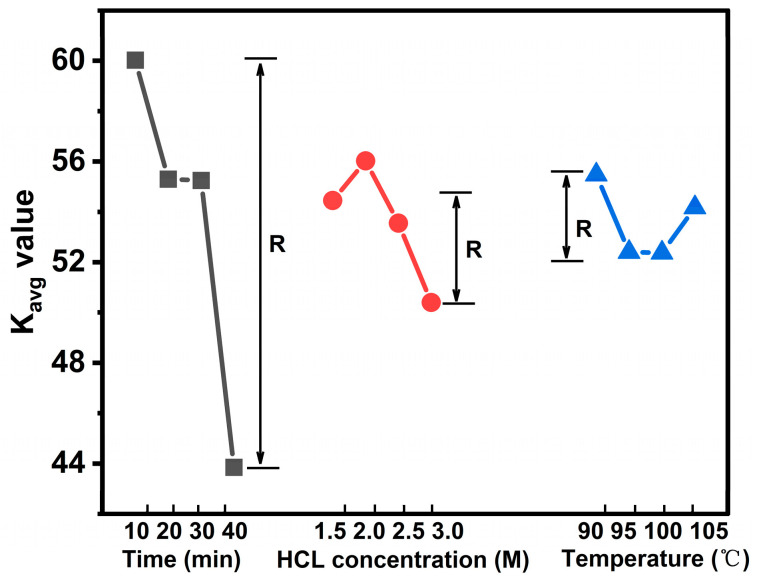
K_avg_ value plot of factors at different levels.

To optimize the hydrolysis conditions, an orthogonal experimental design was employed, considering HCl concentration, reaction time, and temperature. The range analysis results, summarized in Table 3, indicate that these factors influenced MCC yield to varying degrees. Among them, reaction time had the most significant impact, followed by HCl concentration and temperature according to the R value. In addition, according to the calculation results of K and K_avg_ values, the optimal hydrolysis condition corresponding to the optimal level is a 2.0 M HCl concentration, 90 °C, and 10 min of reaction time. Among them, K and K_avg_ values represent the total and average yield of all different levels for each factor, and the optimal level is the experimental condition corresponding to the maximum K_avg_ value. This trend is further illustrated in Figure 1. To assess the impact of hydrolysis time on yield, a multifactor analysis of variance was conducted (Table 4). The results revealed that hydrolysis time had the most significant effect on MCC yield (*p* = 0.0001), followed by HCl concentration and temperature. Prolonging the material’s exposure to acid is anticipated to have a pronounced effect on cellulose, as the β-1,4-glucosidic bonds are highly susceptible to acid hydrolysis [31]. According to previous works, increasing the contact time of material with acid is expected to have an intensive influence on cellulose, because the β-1,4-glucosidic bonds of cellulose is sensitive to acid. So, the findings of this study align with conclusions reported in previous research [19,32].

### 3.2. FTIR Spectroscopy

**Figure 2:**
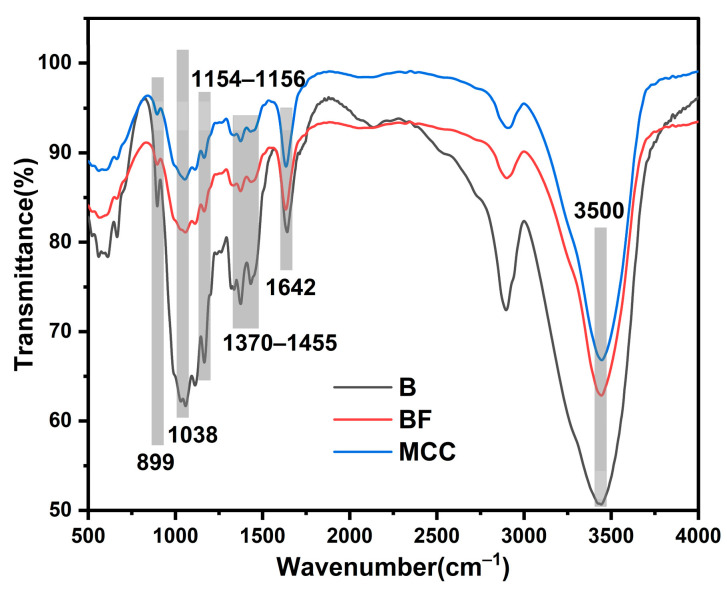
FTIR spectra of raw bamboo, bamboo fiber and MCC.

Figure 2 presents the FTIR spectra of raw bamboo powder, BF, and MCC. The absorption peak around 899 cm^−1^ corresponds to the glucosidic bond. A slight decrease in its intensity suggests that the fundamental chemical structure of MCC derived from the acid-base treatment of BF remained largely unchanged. The absorption peak at approximately 1038 cm^−1^ corresponds to the C-O stretching vibration of alcohol hydroxyl groups in cellulose, while the peak at 1642 cm^−1^ is associated with the stretching vibration of glucose lactone. The absorption peak near 2900 cm^−1^ represents the C-H stretching vibration, and the broad peak around 3500 cm^−1^ corresponds to the stretching vibration of hydroxyl (-OH) groups in cellulose. The FTIR spectra of all three samples exhibited high similarity, indicating that BF and MCC retained the same infrared functional groups as raw bamboo. Compared to raw bamboo, the O-H stretching vibration absorption band in BF and MCC shifted to a lower frequency with reduced intensity, suggesting that acid/alkali treatment partially disrupted intramolecular hydrogen bonds while strengthening intermolecular hydrogen bond interactions. The peak corresponding to the -C-O-C- stretching of the β-1,4-glycosidic linkage in cellulose appeared at 1154 cm^−1^ in BF and 1156 cm^−1^ in MCC. The reduced intensity of this band in the MCC spectrum may be attributed to the presence of non-cellulosic constituents. Although the chemical groups of BF and MCC have not changed compared to natural bamboo, their content will undergo significant changes during the isolation process. The relative intensities of bands related to -CH, -CH_2_ vibrations and to aromatic ring modes (1370–1455 cm^−1^) decreased, indicating lignin degradation. Combined with component analysis, it can be seen that the lignin content of BF and MCC has significantly decreased, while also retaining some hemicellulose. The MCC content reached 69.3%, as shown in Table 6.

### 3.3. XRD Analysis

**Figure 3:**
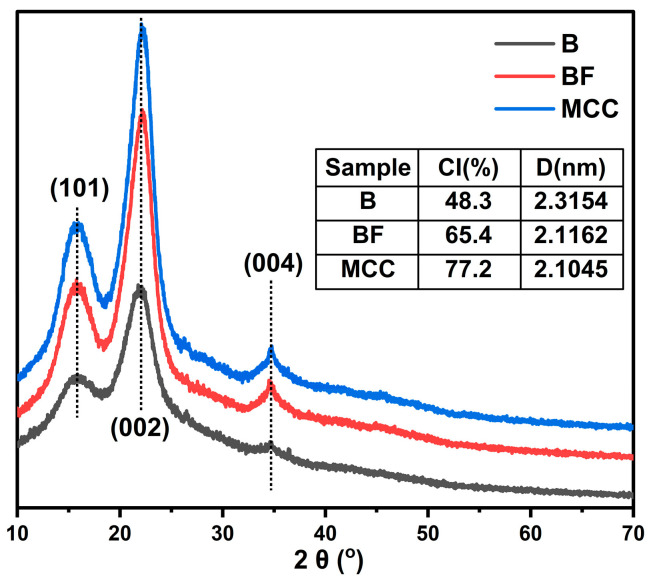
XRD spectra, crystallinity and crystalline region size of raw bamboo, bamboo fiber and MCC.

As shown in Figure 3, the diffraction peak positions of raw bamboo, BF, and MCC remained largely unchanged. Two prominent characteristic peaks were observed at approximately 15° and 22° (2θ), corresponding to the (101) and (002) crystal planes of cellulose I, along with a smaller peak near 34°, associated with the (004) plane. These results indicate that the isolation of BF did not alter the crystalline structure of cellulose, and the MCC obtained through acid treatment retained the cellulose I crystal form with the same crystal cell structure. Bamboo powder contains significant amounts of lignin and hemicellulose, contributing to a high proportion of amorphous regions and resulting in a relatively low CI of 48.3%. However, the removal of soluble components and partial hemicellulose through alkali treatment and delignification facilitates the rearrangement of cellulose molecules in BF, MCC and the grain size of the crystalline region decreased. While the intensity of both crystalline and amorphous diffraction peaks decreased, the overall crystallinity of BF and MCC showed a marked improvement. The enhanced crystallinity of MCC after acid treatment is primarily attributed to the penetration of hydrogen ions into the amorphous regions of cellulose, accelerating the cleavage of glycosidic bonds and increasing the proportion of crystalline cellulose. However, the CI of MCC was not 100%, indicating the presence of residual amorphous regions. This suggests that MCC retains a coexistence of both crystalline and amorphous regions.

Table 5 shows the crystallinity of MCC prepared from different raw materials. From previous research results, it can be seen that the crystallinity of MCC prepared from bamboo is distributed between 71.82% and 78.6%, which is similar to the crystallinity of MCC prepared in this study. In addition, kenaf with high fiber content can achieve a crystallinity of over 90, while some biomass waste materials have lower crystallinity. Overall, using bamboo as the raw material source for MCC has a relatively high crystallinity and can be used as a raw material for large-scale industrial production of MCC.

### 3.4. Thermal Analysis

**Figure 4:**
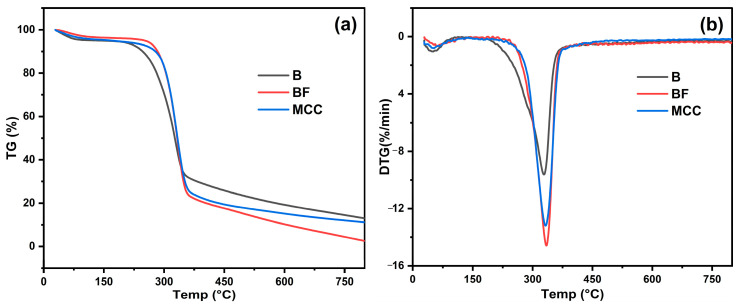
TG (**a**) and DTG (**b**) curves of raw bamboo, bamboo fiber and MCC.

Both TG and DTG are commonly used to investigate the thermal and degradation properties of samples. Due to the chemical structural differences between hemicellulose, cellulose, and lignin, they typically decompose at different temperatures. Numerous studies on the thermal decomposition of lignocellulosic materials have reported that cellulose begins to decompose at around 315 °C, continuing up to 400 °C. The maximum weight loss rate for cellulose occurs at around 350 °C, with only 6.5% of the material remaining at 400 °C. Hemicellulose begins to degrade at approximately 220 °C and continues until 315 °C, with the maximum weight loss rate occurring at 268 °C. Lignin, on the other hand, has a relatively wide decomposition range, with a final solid residue of about 46% between 200 and 700 °C. Figure 4 presents the TG and DTG curves for raw bamboo, BF, and MCC. Below 100 °C, a minor degradation peak is observed in raw bamboo and BF, primarily due to the evaporation of adsorbed water. The presence of various impurities in raw bamboo results in an earlier onset of degradation, around 200 °C, corresponding to the decomposition of pectin, lignin, and other non-cellulosic components. The maximum weight loss rate occurs at approximately 285 °C, indicating the degradation of cellulose. With the removal of impurities, BF exhibits a higher degradation temperature compared to raw bamboo. The TG curves of MCC exhibit two distinct degradation stages, reflecting the sample’s weight loss. The initial stage, occurring between 60 and 140 °C, corresponds to the evaporation of bound water within the cellulose. The second stage, observed in the range of 250–450 °C, involves dehydration, decarboxylation, depolymerization, and the decomposition of glycosyl units, ultimately leading to char residue formation.

A comparison of the thermogravimetric curves of BF and MCC reveals that while their thermal decomposition temperatures are similar, MCC exhibits a lower residual mass. This suggests a reduction in thermal stability, likely due to two main reasons for this. Firstly, acid hydrolysis reduces particle size, increases nanostructures and specific surface area and exposes free molecular chain ends on the surface. Secondly, during the hydrolysis process, cellulose chains are damaged and broken, resulting in the adsorption of many small molecules and breakage points on the surface of MCC. In addition, the irregular and non-compact arrangement forms a large number of defect points, which will degrade first at lower temperatures. Those structural changes enhance reaction activity, leading to more intense decomposition of MCC.

### 3.5. SEM Analysis

**Figure 5:**
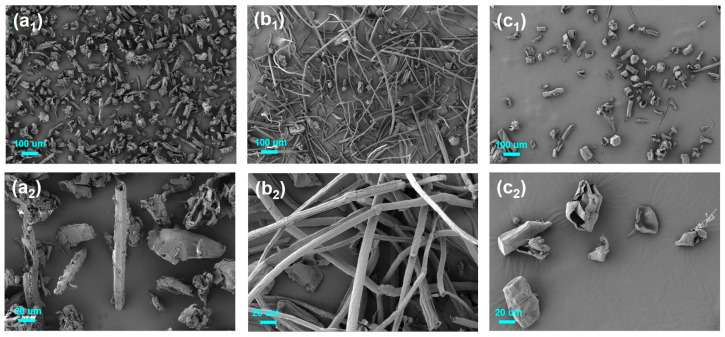
Scanning electron micrographs of raw bamboo (**a_1_**,**a_2_**), bamboo fiber (**b_1_**,**b_2_**) and MCC (**c_1_**,**c_2_**).

**Figure 6:**
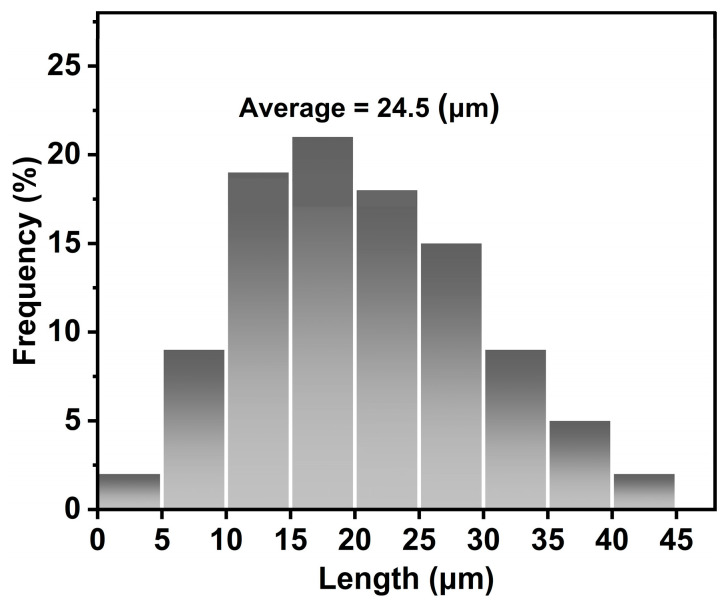
Particle size distribution of the resulting MCC.

The morphologies of raw bamboo, BF, and MCC were analyzed using SEM, with the corresponding images shown in Figure 5. SEM observations revealed evident changes in fiber structure, including changes in shape and size as the materials were processed from raw bamboo to BF and then to MCC. After treatment with toluene/ethanol, the raw bamboo powder retained much of the original bamboo tissue, including fibers and thin-walled cells. However, BF exhibited a smoother surface, suggesting the successful removal of lignin, hemicellulose, and other impurities during processing. When compared to BF, MCC displayed a more irregular structure with micro-sized fibrils and a rougher surface. Most MCC particles were found to have an irregular rod shape with rough surfaces, which aligns with findings from previous studies [26,39]. Previous studies with similar morphological observations indicate that acid hydrolysis imparts a rough surface to MCC, a feature that facilitates the isolation of nanocrystals [22]. In addition, there is not much difference in the MCC morphology as compared with several types of sources such as MCC from Alfa fiber [39], soybean hulls [40], belulang grass [41], oil palm [42], and bamboo [24]. The SEM image of MCC revealed fibers with an average width of 24.5 μm, as depicted in Figure 6. Also, the SEM revealed that BFs have diameters ranging from 20 to 50 µm while MCC filaments present analogous diameters. However, in terms of length, the length of MCC (20 to 100 µm) is significantly smaller than that of BF (>500 µm), which also leads to lower length-to-width ratios, mainly because acid hydrolysis destroys its amorphous region. Overall, the SEM analysis validated the effective conversion of bamboo into MCC with micro-sized particles.

### 3.6. Compositional Analysis

Table 6 shows the results of the compositional analysis of the raw bamboo material samples, BF and MCC and the M_w_, M_n_ and polydispersity (M_w_/M_n_), which were also were summarized. It is apparent that there was a reduction in molecular weight on MCC. Hydrolysis breaks glycosidic bonds in cellulose, causing a decrease in molecular weight and producing MCC with a smaller chain length than the original polymer. In addition, from the results of the component analysis, it can be seen that the lignin content of bamboo is significantly reduced after fiber extraction and MCC has the highest cellulose content.

**Table 6:** Compositional analysis and GPC of raw bamboo, BF and MCC.

Sample	M_n_	M_w_	DP_I_	DP_n_	DP_w_	Cellulose	Hemicellulose	Lignin
MCC	27,819	153,004	5.50	54	295	69.3%	12.2%	6.3%
BF	63,336	380,020	6.00	122	732	57.4%	16.7%	8.0%
Raw bamboo	-	-	-	-	-	39.3%	20.5%	32.4%

M_n_, number-average molecular weight; M_w_, weight-average molecular weight; DP_I_, polydispersity index (M_w_/M_n_); DP_n_, number-average degree of polymerization; DP_w_, weight-average degree of polymerization.

## 4. Conclusions

In this study, MCC was effectively extracted from bamboo fiber through sequential dewaxing, delignification and acid hydrolysis. The impact of hydrolysis parameters was assessed, determining the optimal conditions as 2.0 M HCl at 90 °C for 10 min. SEM analysis revealed that MCC had an irregular rod-like structure with rough surfaces, whereas BF displayed a smoother morphology. XRD analysis confirmed the presence of the cellulose I polymorph structure and showed an evident increase in crystallinity (77.2%) in the acid-hydrolyzed BF compared to the raw material. FTIR results indicated that BF and MCC retained the same functional groups as raw bamboo. TG analysis showed a slight reduction in MCC’s thermal stability. These findings highlight the efficiency of the applied method in extracting MCC from raw bamboo.

Future research on bamboo-derived microcrystalline cellulose could focus on optimizing scalable, eco-friendly production methods to reduce chemical waste and energy consumption and designing scalable continuous production systems for high-yield. Furthermore, expanding applications in biodegradable composites and pharmaceuticals, enhancing functionality through nanostructure engineering, and validating its sustainability and cost-effectiveness to establish it as a viable alternative to conventional sources like cotton could also be applicable in the future.

## Figures and Tables

**Table 1:** Factors and levels of the orthogonal experiment.

Level	Factors		
	HCl Concentration (M)	Temperature (°C)	Time (min)
1	1.5	90	10
2	2.0	95	20
3	2.5	100	30
4	3.0	105	40

**Table 2:** Three factor–four level test.

Experiment Number	HCl Concentration (M)	Temperature (°C)	Time (min)
(1)	1	1	1
(2)	1	2	2
(3)	1	3	3
(4)	1	4	4
(5)	2	1	2
(6)	2	2	1
(7)	2	3	4
(8)	2	4	3
(9)	3	1	3
(10)	3	2	4
(11)	3	3	1
(12)	3	4	2
(13)	4	1	4
(14)	4	2	3
(15)	4	3	2
(16)	4	4	1

**Table 3:** Range Analysis.

Term	Level	Temperature (°C)	HCl Concentration (M)	Time (min)
K value	1	221.9	217.8	240.1
	2	209.6	224.1	221.2
	3	209.5	214.2	221
	4	216.7	201.6	175.4
K_avg_ value	1	55.48	54.45	60.03
	2	52.4	56.03	55.3
	3	52.38	53.55	55.25
	4	54.17	50.4	43.85
Optimal level		1	2	1
R		3.1	5.63	16.18

**Table 4:** Multivariate analysis of variance.

	Sum of Squares	df	Mean Square	F	*p*
Intercept	45,978.081	1	45,978.081	5080.568	0.000 **
HCl concentration	67.382	3	22.461	2.482	0.158
Temperature	27.147	3	9.049	1	0.455
Time	567.822	3	189.274	20.915	0.001 **
Residual	54.299	6	9.05		

R^2^ = 0.924, ** *p* < 0.01.

**Table 5:** Comparison of CI with other sources discussed in the literature.

Microcrystalline Cellulose	CI (%)	Ref.
Bamboo	78.0	[26]
Cotton stalk	80.29	[33]
Hardwood	80.33	
Softwood	81.56	
Bamboo	78.60	
Sisal	81.13	
Bamboo	71.82	[21]
Bamboo Pulp	75.64	[19]
Wine waste	32.45	[34]
	22.60	
	34.76	
	28.21	
Douglas fir	71	[28]
Rice straw	68.2	
Potato tuber	66.1	
Kenaf fiber	92.2	[35]
Rice husk	77.8	[36]
Wheat bran	43.93	[37]
Oil palm trunk slabs	60.81	[38]

## Data Availability

The original contributions presented in this study are included in the article. Further inquiries can be directed to the corresponding author.
